# Surgical outcomes in canine lymphoplasmacytic stomatitis: a retrospective study of 42 dogs (2012–2022)

**DOI:** 10.3389/fvets.2025.1681133

**Published:** 2025-12-09

**Authors:** Jordan Ford, Cynthia Bell, Taryn A. Donovan, Django Martel

**Affiliations:** 1 Prism Veterinary Dentistry, New York, NY, United States; 2 Specialty Oral Pathology for Animals, Geneseo, IL, United States; 3 Anatomic Pathology Service, Schwarzman Animal Medical Center, New York, NY, United States; 4 Dentistry and Oral Surgery Service, Schwarzman Animal Medical Center, New York, NY, United States

**Keywords:** canine stomatitis, CCUS, CUPS, contact lesions, oral medicine, veterinary dentistry

## Abstract

Lymphoplasmacytic oral mucosal inflammation (stomatitis) in canine patients is common and non-specific. Contact lesions (CL) secondary to periodontal disease are particularly common. Dogs with CL may or may not have canine chronic ulcerative stomatitis (CCUS), yet clinicians are often faced with making treatment decisions without a clear diagnosis. Some may expect canine patients to be treated similarly to feline chronic gingivostomatitis patients, for whom partial- to full-mouth extractions remain the primary intervention. Because this assumption may lead to inappropriate care, clear recommendations for surgical management are needed for canine patients. This retrospective review (2012–2022) evaluated 42 dogs with lymphoplasmacytic stomatitis treated surgically at a dental specialty service. We hypothesized that only a minority of these patients require full-mouth exodontia. Patients were initially stratified as CL or CCUS based on lesion distribution and radiographic findings and reclassified based on treatment response. Clinical outcomes were categorized as complete, partial, or absent response to treatment(s), including COHAT, salvage exodontia, rescue medical management, chronic immunosuppression, and home oral hygiene. Associations between demographic and clinical variables with surgical and clinical endpoints were evaluated using descriptive statistics and chi-square analyses. Small breeds (median 5.4 kg) predominated and were significantly more likely to reach edentulism for any reason. Buccal lesions adjacent to maxillary canine and fourth premolar teeth were most common, but over 60% of cases had mucosal changes beyond the classic paradental lesions. Ultimately, 31% were confirmed as CL and 50% as CCUS. Only 24% underwent salvage exodontia, and 17% were rendered edentulous for mucosal disease management. Achieving edentulism was more common in CL cases (54%) compared to CCUS cases (33%). Most dogs (81%) achieved complete resolution, primarily through treatment of periodontal disease (62%). CL cases showed high response rates to periodontal therapy (83%), while a subset of CCUS patients underwent salvage exodontia (20%) or immunosuppression (5%). The majority of dogs with lymphoplasmacytic stomatitis, including many diagnosed as CCUS, respond to periodontal disease management without full-mouth extractions. These findings support a conservative, staged treatment approach and underscore the need for standardized diagnostic criteria in canine oral inflammatory disease.

## Introduction

For veterinary practitioners in primary care and specialty dentistry, management of canine patients with chronic, lymphoplasmacytic oral mucosal inflammation (chronic lymphoplasmacytic stomatitis) can be frustrating in the midst of evolving clinical terminology and treatment recommendations. In contrast, the most common form of lymphoplasmacytic stomatitis in cats, feline chronic gingivostomatitis (FCGS), has a more widely recognized body of foundational research with relatively consistent treatment guidelines. The current primary intervention for FCGS is partial (i.e., all premolar and molar teeth) to full-mouth extractions followed by immunosuppression with systemic cyclosporine therapy for most patients refractory to exodontia (1–5). Chronic, lymphoplasmacytic stomatitis in the dog is similar in some respects, with both species experiencing potentially debilitating pain from oral inflammatory lesions. However, investigations regarding nomenclature, classification, etiopathogenesis, and optimal treatment paradigms for canine patients are ongoing (6–10).

As early as 1905, various forms of stomatitis were described in multiple domestic veterinary species, including ulcerative gingivitis in dogs progressing to involve the buccal and lingual mucosa (11). Some of the earliest contemporary entries describing canine stomatitis emphasized characteristic buccal mucosal ulcers (“kissing lesions”) apposing the maxillary canine and fourth premolar teeth, formed at points of oral mucosal contact with plaque and calculus-laden dental surfaces (12–14). Accordingly, the descriptor chronic ulcerative paradental stomatitis (CUPS) was proposed and popularized (6, 12–16). Treatment recommendations at that time focused on fastidious plaque control via consistent home oral care and regular professional periodontal disease treatment, including removal of supra- and sub-gingival dental deposits and dental extractions as indicated by stage of periodontal disease. Aggressive surgical intervention (i.e., partial- to full-mouth extractions, regardless of periodontal health) was considered for refractory or severely affected cases (6, 12–15). Breeds including the Maltese, Cavalier King Charles Spaniels, and Scottish Terriers were reported to be predisposed (12–16); however, these breeds were not well represented in later research, and some have questioned whether earlier reports were conflating CUPS with eosinophilic oral disease, where such breed overrepresentation has been more consistently reported (7, 8, 17, 18). Mucosal biopsy is required to rule out competing differential diagnoses for erosive and ulcerative oral lesions in canine patients, including autoimmune disease (e.g., lupus erythematosus, pemphigus vulgaris, mucous membrane pemphigoid, erythema multiforme), uremia, mucosal drug reaction, thermal or chemical trauma, infection, or neoplasia (e.g., epitheliotropic T-cell lymphoma) (7, 19–23).

Within the past several years, important research has sought to more rigorously define and characterize canine chronic ulcerative stomatitis (CCUS), including investigation of pathogenesis and treatment guidelines (8–10). More robust patient sampling has found small (<10 kg) dogs, castrated males, and terrier breeds to be overrepresented (8). The pathogenesis remains incompletely understood, but there is evidence that CCUS is a T lymphocyte-mediated inflammatory disease process (8, 9). Three histologic subtypes of CCUS (granulomatous, lichenoid, and deep) have been proposed (9). While the granulomatous subtype correlated with more severe disease (9), the histologic subtypes have not yet been explained by differences in pathoetiology. As some study populations had approximately 40% of mucosal lesions associated with edentulous areas (i.e., free of dentition and therefore not “paradental”), further use of the earlier CUPS terminology as a distinct clinical entity has been discouraged (9).

As CUPS is eliminated from usage, clear definitions for preferable terminology are important. CCUS is an appropriate clinical diagnosis for many dogs that would have previously been considered to have CUPS. However, CCUS is not perfectly synonymous with CUPS. For example, a clinical diagnosis of CCUS may be inappropriate for solitary contact-associated lesion that would have historically been called CUPS. The authors propose “contact lesion” (CL) as useful terminology to refer to secondary mucosal changes resulting from contact with adjacent areas of bacteria-mediated inflammation (e.g., areas of severe calculus deposition, advanced periodontitis, exposed necrotic bone) and responding favorably to management of the primary, local disease (7). The distinction between CLs and CCUS is largely based on clinical findings and response to therapy (24). Histopathology is not specific since both types of lesion commonly have lymphoplasmacytic inflammation with erosion or ulceration of the mucosal epithelium (7). Contact lesions, if secondary to overt periodontitis, would be more responsive to reduction of plaque bacteria and appropriate exodontic intervention.

The extent and severity of inflammation in CCUS cases are often disproportionate to the amount of plaque or calculus deposition, and aggressive plaque control is not considered to be an effective management strategy (8). Systemic anti-inflammatory protocols [e.g., pentoxifylline and tocopherol (PENTO), subantimicrobial doxycycline (SAD, 2 mg/kg PO daily)] are increasingly described in veterinary dentistry (25, 26), but only recently has a clinical trial supported a combination of cyclosporine and metronidazole as a much-needed treatment option for CCUS (10). A single case report has been published describing the use of adipose-derived mesenchymal stem cells for local treatment of CCUS (27). Overall, successful management of CCUS may require medical therapy combined with targeted surgical treatment of periodontal disease (8–10, 24).

Whether in the primary or referral dentistry setting, chronic lymphoplasmacytic stomatitis remains a complex diagnosis in canine patients. Chronic stomatitis in dogs is not a singular diagnosis and, in the absence of a definitive diagnosis, the clinician and client may be left without clear expectations for treatment or prognosis. In the authors’ experience, if stomatitis has been discussed prior to referral, many clients present their dogs anticipating full-mouth extractions as the primary intervention. Aversion created by this misconception could delay needed treatment, increasing patient morbidity. This retrospective study was performed to challenge the perception of full-mouth exodontia as the primary intervention and to establish outcome metrics for the purposes of client discussions. The primary aim was to retrospectively describe the response of canine lymphoplasmacytic stomatitis to surgical and medical treatment. Secondary aims included the following: (1) to determine what proportion of dogs with only paradental CL have resolution of mucosal inflammatory lesions following periodontal disease management, (2) to determine what proportion of dogs with presumptive CCUS have resolution of mucosal inflammatory lesions following periodontal disease management, (3) to determine what proportion of dogs with presumptive CCUS have persistent lesions following periodontal disease management but resolution of mucosal inflammatory lesions following salvage exodontia, and (4) to determine what proportion of dogs with presumptive CCUS have refractory mucosal inflammatory lesions following salvage exodontia. Based on clinical practice experience, our hypothesis was that only a minority of canine stomatitis cases require full-mouth exodontia.

## Materials and methods

### Case selection

Archives of the Anatomic Pathology Service and medical records of the Dentistry and Oral Surgery Service of the Schwarzman Animal Medical Center were queried from 2012 to 2022 for dogs with a clinical diagnosis of CUPS or an oral mucosal biopsy supportive of stomatitis. The respective medical records from the Dentistry and Oral Surgery Service were reviewed. Inclusion required biopsy of oral mucosa with a histopathologic diagnosis of lymphoplasmacytic stomatitis with or without mention of mononuclear or neutrophilic inflammation. Cases with biopsy reports concerning for primary eosinophilic, autoimmune, or neoplastic disease were excluded. A board-certified veterinary pathologist performed histopathology either within the research institution (TAD) or by an outside laboratory when samples were unavailable for review. All biopsy reports from an outside laboratory were reviewed by at least one of the authors (JF, TAD) to ensure that the diagnosis and biopsy site were appropriate for inclusion. Case inclusion also required a complete medical record with at least one follow-up examination (either conscious or anesthetized) within the authors’ institution after initial comprehensive oral health assessment and treatment (COHAT). Board-certified veterinary dentists or clinicians under their supervision managed the cases within their department. Given the retrospective nature of the study, ethics committee review was not required.

### Data collection

The following patient data was extracted from baseline assessment: age, breed, sex, neuter status, body weight, presenting complaint(s), pre-anesthetic blood work abnormalities, and comorbid disease processes. Records for each COHAT were reviewed for anesthetized examination findings (i.e., lesion distribution), staging of periodontal disease (i.e., dental charting findings, intraoral radiography interpretation), and exodontic intervention. Mucosal lesion categories were as follows: buccal mucosa paradental to the maxillary canine teeth (104/204), buccal mucosa paradental to the maxillary fourth premolar through first molar teeth (108–109/208–209), buccal mucosa paradental to the mandibular canine teeth (304/404), buccal/vestibular mucosa paradental to the maxillary incisor teeth (101–103/201–203), buccal mucosa paradental to the mandibular first molar teeth (309/409), generalized buccal mucosa, lingual or sublingual mucosa, oral commissures, hard or soft palatal mucosa, and palatoglossal folds.

The number of COHATs per patient and the number of days between anesthetized assessments were calculated. The number of extractions, if performed, was recorded for each COHAT, with the distinction made between those due to advanced periodontitis (i.e., periodontal disease stages three to four) versus salvage extraction of teeth solely to relieve contact with the oral mucosa. The number of patients rendered edentulous during the study period was calculated. The cumulative medical records for each patient, representing subsequent conscious and anesthetized assessments as available across departments within the residency institution, were reviewed for response to treatment, which was categorized as complete (i.e., resolution of oral lesions and clinical signs), partial (i.e., persistent but improved oral lesions and/or clinical signs at last available examination), or no response. Given the retrospective nature of the study, standardized measures for improvement of mucosal lesions and clinical signs were unavailable. Treatment response was categorized in part based on clinician notes within the medical and dental records describing changes to mucosal lesion size, intensity, and distribution. Changes in observed clinical signs were based on client report and treating clinician notes (e.g., improved comfort on conscious oral examination, if noted).

Home oral hygiene (e.g., tooth brushing, dental chews, antiseptic oral rinses) was recommended for every patient, and any home interventions reported by the clients were noted when recorded in the available medical records. During the study period following baseline COHAT at the authors’ institution, patients at times required short-term rescue medical management (e.g., antibiotics, analgesics, and/or short tapering courses of corticosteroids) prescribed either by their primary veterinarians or colleagues within the authors’ department. Given the heterogeneity in drug selection, dosing, and duration amongst clinicians, these supportive therapies were broadly categorized as follows: antibiotics, NSAIDs, gabapentin, opioids, and tapering corticosteroids. Chronic medical management (i.e., immunomodulation or immunosuppression greater than 2 weeks), though not commonly employed within the department at the time, was noted when applicable.

### Data analytic strategy

All analyses were conducted in SPSS V23. Descriptive statistics were used to summarize patient demographics, number of comprehensive treatments, oral mucosal lesion distribution, extent of exodontia, and treatment response. We report medians (interquartile range [IQR]) for non-normal data and means (standard deviation [SD]) for normally distributed data. Frequencies or proportions were used to summarize categorical data. For initial patient stratification, those dogs with only paradental mucosal lesion distribution and with concurrent radiographic evidence of periodontal attachment loss were preliminarily diagnosed as CL cases. Patients with non-paradental mucosal lesions and/or those without radiographic evidence of periodontitis were preliminarily designated as CCUS cases.

Spearman’s rho (𝜌) correlation coefficients describe the strength and direction of associations between the following non-normally distributed variables: the sum of lesion distribution sites, the total number of the anesthetized treatments, and the total number of extractions during the study period. To evaluate associations between dichotomous variables, we used Chi-square and Fisher’s Exact tests, which do not rely on assumptions about the underlying population distribution being normal. First, Chi-square analyses^1^ within SPSS’s Custom Tables module were used to compare demographics, clinical characteristics, and preliminary diagnosis across three surgical outcomes: dogs with and without full-mouth extractions for any reason; dogs with and without any salvage extractions due to stomatitis; dogs with and without full-mouth extractions due to stomatitis. Second, all patient characteristics, preliminary diagnosis, and surgical outcomes were compared across clinical outcome: complete, partial, or absent. A two-tailed alpha of 0.05 was used to adjudicate statistical significance in all analyses. Given the exploratory nature of this retrospective study and the limited sample size, no formal adjustment was applied for multiple comparisons (e.g., Bonferroni correction) as this can inflate Type II error and obscure potentially meaningful trends. As such, results should be interpreted cautiously as the potential for inflated Type I error is present. All *p*-values are presented to guide interpretation and further hypothesis generation.

Finally, patients were reclassified from initial stratification based on response to routine management of periodontal disease, short-term medical management, chronic medical management, and salvage exodontia. Patients with only paradental lesion distribution and complete resolution with routine periodontal disease management were confirmed as cases of CL secondary to periodontal disease. Patients achieving complete resolution with rescue medical management, chronic immunosuppression, and/or salvage exodontia were confirmed as CCUS cases. Patients with no or partial clinical response during the study period were classified as having no definitive diagnosis.

## Results

### Patient characteristics at presentation

Preliminary archival and medical record queries yielded 60 dogs with histologic diagnoses of chronic stomatitis during the specified time period. Forty-two cases met inclusion criteria, with insufficient follow-up (50%) or incomplete medical records (28%) being the most common reasons for exclusion. Patient age at initial assessment ranged from 1.9 to 15.5 years (mean 9.4; SD 3.0). Patient body weight ranged from 1.7 to 40.2 kilograms (median 5.4; IQR 8.9). Twenty-three patients (55%) were male (17 castrated, six intact), and 19 (45%) were female (18 spayed, one intact). Twenty-two breed categories were represented, with the most common being Maltese (17%), Maltese cross not otherwise specified (7%), Miniature Poodle (7%), Cocker Spaniel (7%), and Dachshund (7%). Full signalment data is shown in Table 1.

**Table 1:** Patient signalment.

Breed	Sex status	Age (Y)
Bichon Frise	FS	13.8
Cavalier King Charles Spaniel	MC	8.8
Chihuahua	FS	10.1
Chihuahua	FI	10.2
Cocker Spaniel × Poodle Cross	MC	10.9
Cocker Spaniel × Poodle Cross	MC	5.8
Cocker Spaniel	MC	12.2
Cocker Spaniel	MC	3.9
Cocker Spaniel	MI	8.8
Cross NOS	MC	9.0
Cross NOS	MI	6.0
Dachshund	FS	13.9
Dachshund	MC	9.4
Dachshund	MI	10.2
English Bulldog	MC	9.4
Havanese	FS	15.5
Havanese	FS	9.6
Labrador Retriever	FS	5.9
Labrador Retriever	MC	11.1
Maltese	FS	7.2
Maltese	FS	11.0
Maltese	MC	10.3
Maltese	MC	12.9
Maltese	MC	12.4
Maltese	MI	11.7
Maltese	MI	7.0
Maltese Cross NOS	FS	9.0
Maltese Cross NOS	FS	13.2
Maltese Cross NOS	MC	10.5
Manchester Toy Terrier	MC	1.9
Miniature Poodle	MI	9.8
Miniature Poodle	FS	11.8
Miniature Poodle	FS	7.8
Pomeranian	MC	9.8
Rottweiler	FS	9.4
Samoyed	FS	7.0
Schnauzer	MC	6.0
Silky Terrier	MC	11.2
Tibetan Terrier	FS	7.1
Wheaten Terrier	FS	2.0
Yorkshire Terrier	FS	6.8
Yorkshire Terrier	FS	12.7

Presenting complaints at initial assessment included 37 (88%) patients with halitosis, 31 (74%) with known periodontal disease, 25 (60%) with signs of oral pain (e.g., heady shy behavior, pawing or rubbing the face, altered mastication), six (14%) with hypersalivation, five (12%) with oral hemorrhage, five (12%) with hyporexia, three (7%) with change in dietary preference, two (5%) incidentally noted, one (2%) with lethargy, and just one (2%) patient with a previously identified and biopsied mucosal lesion. Hemogram changes were rare, with three (7%) patients with a monocytosis, two (5%) with a neutrophilia, one (2%) with anemia, and one (2%) with a thrombocytosis. Serum biochemical abnormalities were more consistent, with 14 (33%) dogs having a hyperglobulinemia. Comorbid disease processes were generally uncommon, with myxomatous mitral valve disease (14%), chronic dermatopathy (12%), and chronic enteropathy (7%) being most prevalent within the study population.

### Initial anesthetized assessment

**Figure 1:**
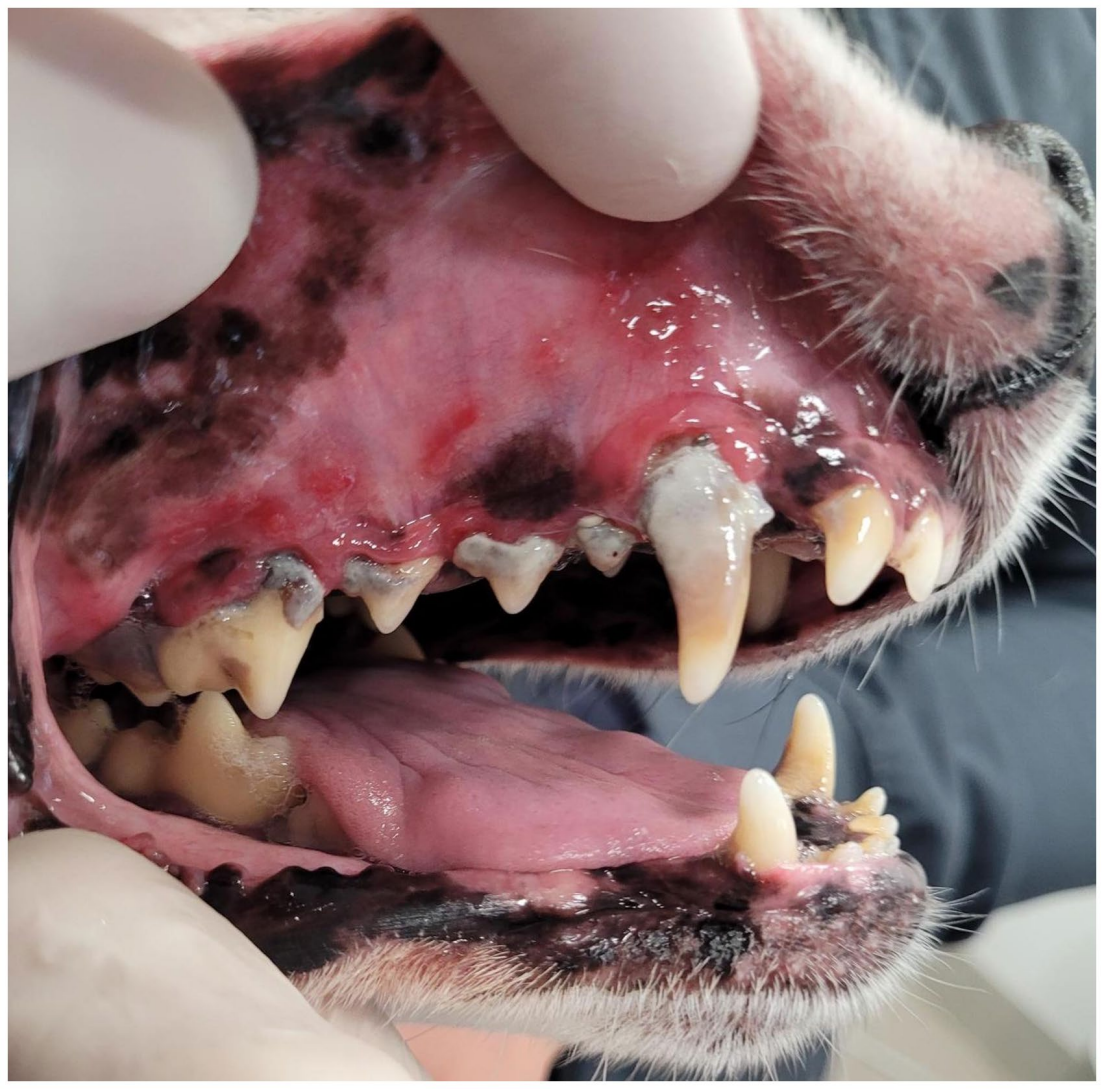
Buccal mucosal ulceration apposing advanced periodontal disease and accumulated foreign material.

On anesthetized oral examination, mucosal lesion site categories in a given patient ranged from one to six (median three). In total, 115 oral mucosal lesion location categories were noted in the study population, with 78 (68%) being paradental buccal or vestibular mucosal changes (Figure 1). The majority of patients (62%) had at least one mucosal lesion outside of the paradental location categories, with only 16 (38%) patients having solely paradental buccal or vestibular mucosal lesions. Buccal mucosal ulcers apposing the maxillary canine (104/204) and fourth premolar through first molar teeth (108–109/208–209) were most common, occurring in 32 (76%) and 23 (55%) dogs, respectively. Lingual or sublingual mucosal lesions were noted in 18 (43%) cases, paradental to the mandibular first molar teeth (309/409) in 12 (29%), paradental to the maxillary incisor teeth (101–103/201–203) in eight (19%), oral commissures in seven (17%), generalized buccal mucosal changes in seven (17%), paradental to the mandibular canine teeth (304/404) in three (7%), palatal mucosal lesions in two (5%), and changes to the palatoglossal folds in two (5%) cases. One patient (2%) had fistulous draining tracts in the mucosa overlying an incisive sequestrum. Of the 16 patients with only paradental mucosal lesion distribution, 12 had concurrent radiographic evidence of periodontal attachment loss; accordingly, these 12 (29%) cases were initially diagnosed as CL. Thirty dogs (71%) were initially classified as presumptive CCUS based on non-paradental mucosal lesion location (*n* = 26) or paradental lesions in the absence of radiographically confirmed periodontitis (*n* = 4).

**Figure 2:**
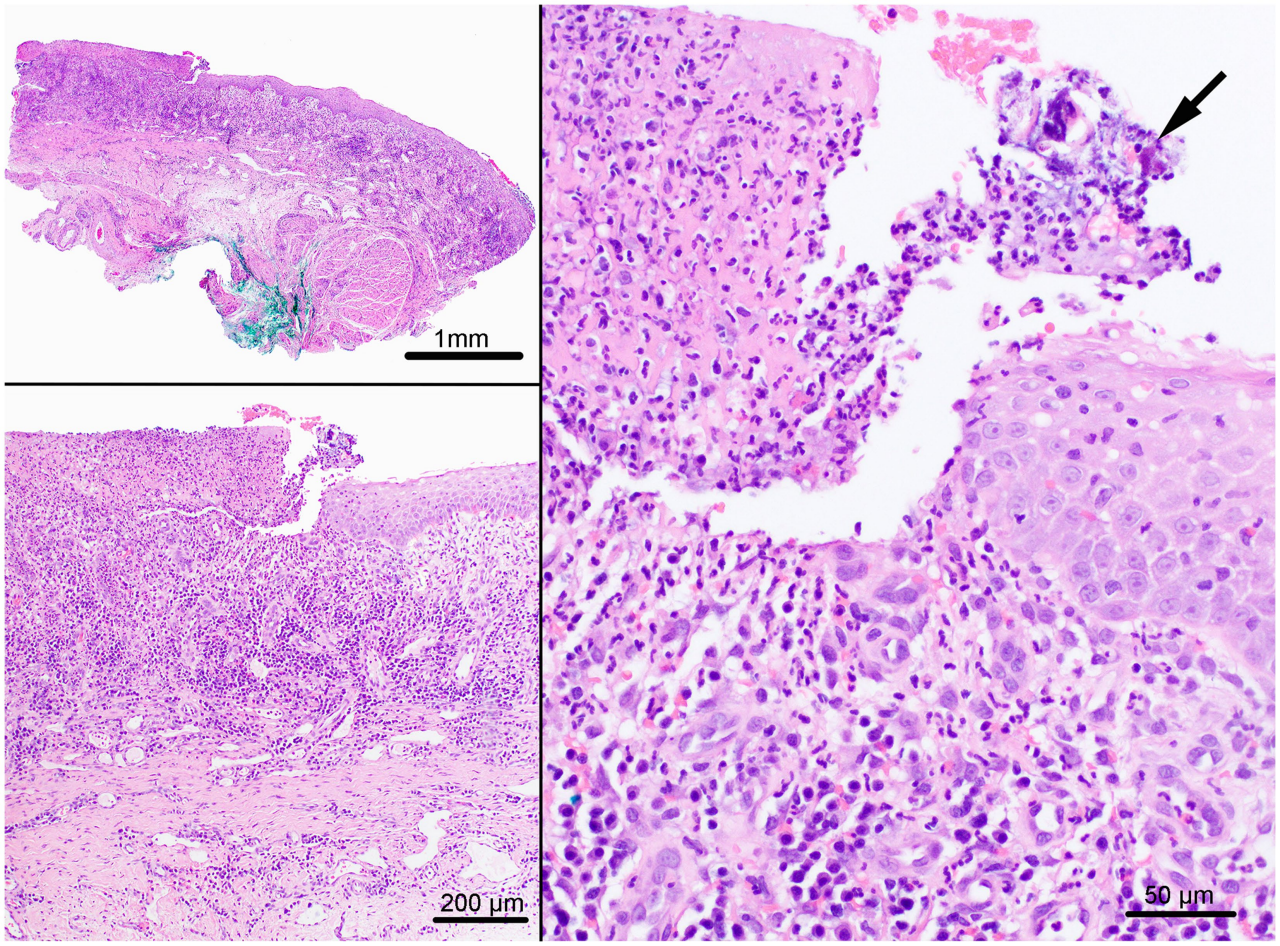
Chronic stomatitis with visible ulceration and bacteria (arrow). Hematoxylin and eosin. Scale bar: 1 mm, 200 um, 50 um.

Aside from confirmation of a previous biopsy diagnosis consistent with chronic stomatitis, quantitative histopathologic reassessment of all cases was beyond the scope of this study. Representative review of paradental and non-paradental lesions found both to have variable degrees of erosion to ulceration and hyperplasia of the epithelium with spongiosis and hydropic degeneration (Figure 2). Inflammation was pleocellular but predominantly lymphoplasmacytic and neutrophilic with some granulation tissue formation when ulceration was present. Both lesion groups had occasional superficial bacteria. Subjectively, superficial spongiosis and hydropic changes to the epithelium were more prominent in the paradental contact lesions.

### Case management and response to therapy

The number of COHATs for each patient within the study period ranged from 1 to 7 (median 1.5; IQR 1) with 21 dogs (50%) undergoing a single anesthetized assessment and treatment. For the 50% of patients undergoing more than one anesthetized assessment, the interval between COHATs ranged from 63 to 844 days (median 240; IQR 242). Total length of follow-up (i.e., first anesthetized assessment to last examination) ranged from 14 to 2,182 days (median 115.5; IQR 507.5).

The total number of teeth extracted per patient ranged from 0 to 42 (median 14.5; IQR 10). Ten (24%) patients had extractions not otherwise indicated due to stage of periodontal disease. Fourteen (33%) patients were rendered edentulous during the study period: seven were due to progression of periodontitis, and seven were salvage procedures to relieve mucosal disease. The total number of lesion site categories was moderately associated with an increase in both the total number of COHATs (𝜌 = 0.50, *p* = 0.002) and the total number of teeth extracted during the study period (𝜌 = 0.36, *p* = 0.019).

During the study period, 17 (40%) patients required rescue medical management at some point after initial COHAT, including 15 (36%) being treated with a systemic antibiotic, nine (21%) receiving an NSAID, five (12%) undergoing tapering corticosteroid courses, and two (5%) each receiving gabapentin or an opioid. Only two patients (5%) underwent chronic immunosuppressive therapy during the study period, one with dentition present and one after having been rendered edentulous. Both were administered chlorambucil in consultation with internal medicine specialists. Only eight (19%) patients reportedly received any form of oral hygiene intervention at home, with six (14%) having daily oral antiseptic rinses, three (7%) having their teeth brushed, and one (2%) utilizing dental wipes.

In total, 34 of 42 (81%) patients achieved complete resolution of oral mucosal lesions and clinical signs during the study period. No cases were categorized as having an absent response to treatment. Of the eight (19%) patients categorized as partial responders (i.e., having no definitive diagnosis), half were lost to follow-up after additional treatment recommendations were made. Of the three patients achieving salvage edentulism with a partial clinical response during the study period, one was lost to follow-up after the seventh anesthetized assessment and treatment; one had persistent lesions and signs that were improved relative to baseline, resulting in the client declining further intervention of any sort; and one had mucosal lesions that improved but did not fully resolve with chlorambucil treatment.

Of the 12 cases preliminarily diagnosed as CL, 10 (83%) had resolution of their mucosal inflammatory lesions following periodontal disease management (i.e., supra- and sub-gingival scaling, extractions due to advanced periodontitis). Two were reclassified after failing to respond to routine management of concurrent periodontal disease (one as confirmed CCUS after complete resolution with salvage exodontia, one as having no definitive diagnosis).

Of the 30 cases preliminarily diagnosed as CCUS, 15 (50%) responded completely to periodontal disease management; six (20%) responded completely to salvage exodontia; one (3%) responded completely with routine periodontal disease management, rescue medical management, and home oral hygiene; and one (3%) achieved a complete response with routine periodontal disease management and chlorambucil administration instead of any salvage exodontia. Ten (33%) presumptive CCUS patients were reclassified. Three cases with only paradental lesions but without radiographic evidence of periodontitis achieved complete resolution with management of concurrent periodontal disease and without rescue medical management or home oral hygiene; accordingly, these cases were reclassified as CL. Seven (23%) preliminarily diagnosed CCUS patients only achieved a partial clinical response during the study period and were therefore reclassified as having no definitive diagnosis.

At the end of the study period, 13 of 42 patients (31%) were confirmed as CL, 21 patients (50%) as CCUS, and 8 (19%) could not be definitively diagnosed. Ten patients (24%) were reclassified from presumptive diagnosis during the study period. Achieving edentulism was more common in patients ultimately designated as CL cases (7 of 13 = 54%) compared to confirmed CCUS cases (7 of 21 = 33%). Patient initial stratification, diagnostic reclassification, and treatment outcome are summarized in Table 2.

**Table 2:** Patient stratification, redistribution, and outcome.

		Original stratification			
		CL	CCUS	Total	
Redistribution based on response to therapy	CL	10	3	13	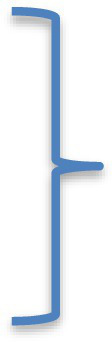 Complete resolution of lesions
	CCUS	1	20	21	
	Lost to follow-up	1	3	4	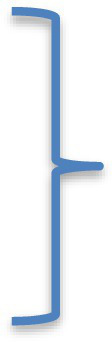 No definitive diagnosis
	Refractory to exodontia	0	4	4	
	Total	12	30	42	

### Correlates of surgical intervention and treatment response

Chi-square and Fisher’s exact tests were conducted to evaluate demographic and clinical correlates of various exodontic outcomes. Table 3 presents comparisons for dogs with and without full-mouth extractions (FME) for any reason. Smaller dogs (<5 kg) were significantly more likely to undergo FME (*χ*^2^(1) = 5.82, *p* = 0.016; Fisher’s exact test *p* = 0.023) for any reason, but no significant associations were observed for sex, number of anesthetized treatments, lesion distribution, preliminary diagnosis, rescue medical management, or home oral hygiene. Table 4 outlines comparisons for dogs with extractions solely due to stomatitis versus those without. Dogs who had salvage extractions due to stomatitis were significantly more likely to have undergone multiple comprehensive treatments as opposed to a single treatment (*χ*^2^(1) = 8.40, *p* = 0.004; Fisher’s exact test *p* = 0.009). There were moderate trends between undergoing any salvage exodontia and having non-paradental mucosal lesions (*χ*^2^(1) = 4.39, *p* = 0.036; Fisher’s exact test *p* = 0.061), as well as requiring rescue medical management between COHATs (*χ*^2^(1) = 4.748, *p* = 0.029; Fisher’s exact test *p* = 0.062), though these relationships ultimately failed to maintain significance. Table 5 compares dogs who became edentulous due to stomatitis versus those who did not. Dogs rendered edentulous due to stomatitis were significantly more likely to have undergone multiple comprehensive treatments (*χ*^2^(1) = 8.40, *p* = 0.004; Fisher’s exact test *p* = 0.009) and to have required rescue medical management between COHATs (*χ*^2^(1) = 7.14, *p* = 0.008; Fisher’s exact test *p* = 0.012). No significant associations were observed for size, sex, lesion distribution, presumptive diagnosis, or home oral hygiene.

**Table 3:** Demographic and clinical comparisons for dogs with and without full-mouth extractions for any reason.

Parameter	Total sample *n* (%)	FME not achieved *n* (%)	FME achieved *n* (%)	*χ* ^2^	Fisher’s exact test (2-sided/1-sided)
Body weight				***χ***^**2**^**(1) = 5.82, *p* = 0.016**	***p* = 0.023/0.018**
<5 kg	19 (45%)	9 (32%)	10 (71%)		
>5 kg	23 (55%)	19 (68%)	4 (29%)		
Sex^a^				*χ*^2^(3) = 0.529, *p* = 0.912	*p* = 1.00/0.545
Female spayed	18 (43%)	12 (43%)	6 (43%)		
Female intact	1 (2%)	1 (4%)	0 (0%)		
Male castrated	17 (41%)	11 (39%)	6 (43%)		
Male intact	6 (14%)	4 (14%)	2 (14%)		
Anesthetized assessments				*χ*^2^(1) = 0.429, *p* = 0.513	*p* = 0.744/0.372
Single	21(50%)	15 (54%)	6 (43%)		
Multiple	21(50%)	13 (46%)	8 (57%)		
Lesion distribution				*χ*^2^(1) = 0.808, *p* = 0.369	*p* = 0.505/0.290
Paradental buccal lesions only	16 (38%)	12 (43%)	4 (29%)		
Paradental and non-paradental lesions	26 (62%)	16 (57%)	10 (71%)		
Presumptive diagnosis				*χ*^2^(1) = 0.000, *p* = 1.000	*p* = 1.000/0.635
CL	12 (29%)	8 (29%)	4 (29%)		
CCUS	30 (71%)	20 (71%)	10 (71%)		
Rescue medical management				*χ*^2^(1) = 2.421, *p* = 0.120	*p* = 0.184/0.111
Yes	17 (40%)	9 (32%)	8 (57%)		
No	25 (60%)	19 (68%)	6 (43%)		
Home oral hygiene				*χ*^2^(1) = 0.077, *p* = 0.781	*p =* 1.00/0.543
Yes	8 (19%)	5 (18%)	3 (21%)		
No	34 (81%)	23 (82%)	11 (79%)		

^a^Fisher’s exact test results are not available for nominal variable with more than two categories, such as sex. Fisher’s exact test results remained non-significant after dichotomizing sex.Results in bold were significant at an alpha level of < 0.05.

**Table 4:** Demographic and clinical comparisons for dogs with and without salvage extractions due to stomatitis.

Parameter	Total sample *n* (%)	No salvage extractions *n* (%)	Salvage extractions *n* (%)	*χ*^2^	Fisher’s exact test (2-sided/1-sided)
Body weight				*χ*^2^(1) = 0.120, *p* = 0.729	*p* = 1.00/0.504
<5 kg	19 (45%)	14 (44%)	5 (50%)		
>5 kg	23 (55%)	18 (56%)	5 (50%)		
Sex^a^				***χ***^**2**^ **(3) = 8.35, *p* = 0.031**	*p* = 0.083/0.068
Female spayed	18 (43%)	16 (50%)	2 (20%)		
Female intact	1 (2%)	1 (3%)	0 (0%)		
Male castrated	17 (41%)	9 (28%)	8 (80%)		
Male intact	6 (14%)	6 (19%)	0 (0%)		
Anesthetized assessments				***χ***^**2**^ **(1) = 8.40, *p* = 0.004**	***p* = 0.009/0.004**
Single	21 (50%)	20 (63%)	1 (10%)		
Multiple	21 (50%)	12 (38%)	9 (90%)		
Lesion distribution				***χ***^**2**^ **(1) = 4.39, *p* = 0.036**	*p* = 0.061/0.038
Paradental buccal lesions only	16 (38%)	15 (47%)	1 (10%)		
Paradental and non-paradental lesions	26 (62%)	17 (53%)	9 (90%)		
Presumptive diagnosis				*χ*^2^(1) = 2.218, *p* = 0.136	*p* = 0.233/0.137
CL	12 (29%)	11 (34%)	1 (10%)		
CCUS	30 (71%)	21 (66%)	9 (90%)		
Rescue medical management				***χ***^**2**^ **(1) = 4.748, *p* = 0.029**	*p* = 0.062/0.038
Yes	17 (40%)	10 (31%)	7 (70%)		
No	25 (60%)	22 (69%)	3 (30%)		
Home oral hygiene				*χ*^2^(1) = 0.008, *p* = 0.930	*p* = 1.00/0.626
Yes	8 (19%)	6 (19%)	2 (20%)		
No	34 (81%)	26 (81%)	8 (80%)		

^a^Fisher’s exact test results are not available for nominal variable with more than two categories, such as sex. After dichotomizing sex, Fisher’s exact test results were significant at an alpha level of 0.01. Results in bold were significant at an alpha level of < 0.05.

**Table 5:** Demographic and clinical comparisons for dogs with and without full-mouth extractions due to stomatitis.

Parameter	Total sample *n* (%)	Not FME due to stomatitis *n* (%)	FME due to stomatitis *n* (%)	*χ* ^2^	Fisher’s exact test (2-sided/1-sided)
Body weight				*χ*^2^(1) = 0.481, *p* = 0.488	*p* = 0.682 /0.388
<5 kg	19 (45%)	15 (43%)	4 (57%)		
>5 kg	23 (55%)	20 (57%)	3 (43%)		
Sex^a^				*χ*^2^(3) = 3.79, *p* = 0.285	*p* = 0.428/0.293
Female spayed	18 (43%)	16 (46%)	2 (29%)		
Female intact	1 (2%)	1 (3%)	0 (0%)		
Male castrated	17 (41%)	12 (34%)	5 (71%)		
Male intact	6 (14%)	6 (17%)	0 (0%)		
Anesthetized assessments				***χ***^**2**^ **(1) = 8.40, *p* = 0.004**	***p* = 0.009/0.004**
Single	21 (50%)	21(60%)	0 (0%)		
Multiple	21 (50%)	14 (40%)	7 (100%)		
Lesion distribution				*χ*^2^(1) = 2.02, *p* = 0.155	*p* = 0.222/0.161
Paradental buccal lesions only	16 (38%)	15 (43%)	1 (14%)		
Paradental and non-paradental lesions	26 (62%)	20 (57%)	6 (86%)		
Presumptive diagnosis				*χ*^2^(1) = 0.840, *p* = 0.359	*p* = 0.651/0.340
CL	12 (29%)	11 (31%)	1 (14%)		
CCUS	30 (71%)	24 (69%)	6 (86%)		
Rescue medical management				***χ***^**2**^ **(1) = 7.14, *p* = 0.008**	***p* = 0.012/0.012**
Yes	17 (41%)	11 (31%)	6 (86%)		
No	25 (60%)	24 (69%)	1 (14%)		
Home oral hygiene				*χ*^2^(1) = 0.494, *p* = 0.482	*p* = 0.601/0.402
Yes	8 (19%)	6 (17%)	2 (29%)		
No	34 (81%)	29 (83%)	5 (71%)		

^a^Fisher’s exact test results are not available for nominal variable with more than two categories, such as sex. Fisher’s exact test results remained non-significant after dichotomizing sex.Results in bold were significant at an alpha level of < 0.05.

Table 6 evaluates demographic and clinical correlates of complete versus partial resolution of lesions and/or clinical signs of stomatitis. Edentulism due to stomatitis (*χ*^2^(1) = 3.09, *p* = 0.079) approached significance in relation to treatment outcome, such that only four of seven (57%) dogs rendered edentulous due to stomatitis achieved complete resolution. Partial responders were significantly more likely to have received rescue medical management between COHATs (*χ*^2^(1) = 4.889, *p* = 0.027; Fisher’s exact test *p* = 0.045). No relationships were found between treatment outcome and patient size, sex, lesion distribution, presumptive diagnosis, number of comprehensive treatments, home oral hygiene, salvage exodontia, and FME for any reason.

**Table 6:** Demographic and clinical comparisons for treatment outcome.

Parameter	Total sample *n* (%)	Complete resolution *n* (%)	Partial resolution *n* (%)	*χ* ^2^	Fisher’s exact test (2-sided/1-sided)
Body weight				*χ*^2^(1) = 0.090, *p* = 0.764	*p* = 1.00/0.534
<5 kg	19 (45%)	15 (44%)	4 (50%)		
>5 kg	23 (55%)	19 (56%)	4 (50%)		
Sex^a^				*χ*^2^(3) = 1.99, *p* = 0.575	*p* = 0.428/0.293
Female spayed	18 (43%)	14 (41%)	4 (50%)		
Female intact	1 (2%)	1 (3%)	0 (0%)		
Male castrated	17 (41%)	13 (38%)	4 (50%)		
Male intact	6 (14%)	6 (18%)	0 (0%)		
Anesthetized Assessments				*χ*^2^(1) = 0.000, *p* = 1.00	*p* = 1.00/0.652
Single	21 (50%)	17 (50%)	4 (50%)		
Multiple	21 (50%)	17 (50%)	4 (50%)		
Lesion distribution				*χ*^2^(1) = 2.75, *p* = 0.098	*p* = 0.127/0.102
Paradental buccal lesions only	16 (38%)	15 (44%)	1 (13%)		
Paradental and non-paradental lesions	26 (62%)	19 (56%)	7 (88%)		
Presumptive diagnosis				*χ*^2^(1) = 1.251, *p* = 0.263	*p* = 0.402/0.257
CL	12 (29%)	11 (32%)	1 (13%)		
CCUS	30 (71%)	23 (68%)	7 (88%)		
Extractions due to stomatitis				*χ*^2^(1) = 1.02, *p* = 0.321	*p* = 0.369/0.280
Yes	10 (24%)	7 (21%)	3 (38%)		
No	32 (76%)	27 (79%)	5 (63%)		
FME due to stomatitis				*χ*^2^(1) = 3.09, *p* = 0.079	*p* = 0.113/0.113
Yes	7 (17%)	4 (12%)	3 (38%)		
No	35 (83%)	30 (88%)	5 (63%)		
FME for any reason				*χ*^2^(1) = 0.077, *p* = 0.781	*p* = 1.00/0.543
Yes	14 (33%)	11 (32%)	3 (38%)		
No	28 (67%)	23 (68%)	5 (63%)		
Rescue medical management				***χ***^**2**^ **(1) = 4.889, *p* = 0.027**	***p* = 0.045/0.036**
Yes	17 (41%)	11 (32%)	6 (75%)		
No	25 (60%)	23 (68%)	2 (25%)		
Home oral hygiene				*χ*^2^(1) = 2.182, *p* = 0.140	*p* = 0.162/0.162
Yes	8 (19%)	5 (15%)	3 (38%)		
No	34 (81%)	29 (85%)	5 (63%)		

^a^Fisher’s exact test results are not available for nominal variable with more than two categories, such as sex. Fisher’s exact test results remained non-significant after dichotomizing sex.Results in bold were significant at an alpha level of < 0.05.

## Discussion

The present study reinforces important prognostic and therapeutic differences between canine and feline patients with lymphoplasmacytic stomatitis. To the authors’ knowledge, this is the first study to describe surgical outcomes for management of chronic stomatitis in canine patients. The established primary intervention for feline patients with FCGS is partial to full-mouth dental extractions, regardless of periodontal health (1–5). The authors have historically managed stomatitis cases primarily with surgery. Extraction of teeth at periodontal disease stages three (25–50% attachment loss) and four (>50% attachment loss) was always recommended, with consideration given to extracting teeth at periodontal disease stages one (0% attachment loss) and two (<25% attachment loss) based on clinical severity and response to therapy. With that in mind, the majority of patients in this study responded favorably to periodontal therapy, and full-mouth extractions were rarely necessary, even in dogs with mucosal ulceration beyond paradental sites. Only 24% of study patients had any extractions for reasons other than periodontal disease stage (i.e., as a salvage strategy for managing mucosal disease). A third of cases were rendered edentulous during the study period; however, half of those were due to progressive periodontitis. Only 17% of patients achieved full-mouth extractions for management of oral mucosal disease, which supports our initial hypothesis. In fact, achieving edentulism was more common in patients ultimately designated as CL cases (54%) compared to confirmed CCUS cases (33%). At minimum, these findings set more realistic expectations for veterinarians and clients faced with an oral mucosal biopsy diagnosis of lymphoplasmacytic stomatitis in a canine patient.

Consistent with previous research, hematologic, serum biochemical, and systemic health disturbances were uncommon and relatively non-specific for canine stomatitis patients. A third of patients had a hyperglobulinemia at presentation, consonant with reports ranging from 21 to 50% (8, 10). Demographically, the present study population resembles those in previous research, with male, middle-aged, small breed dogs predominating (8). The particularly diminutive median body weight of 5.4 kg may be confounded by the densely populated, urban location of the residency institution. The ubiquity of apartment living likely influences breed selection, increasing the popularity of small breed dogs. Selective breeding for smaller stature in dogs has differentially impacted craniofacial development and odontogenesis (6, 28, 29). Smaller breeds of dog generally have dentition that is disproportionately large for their jaw size, resulting in crowding of teeth with already diminished alveolar bone support. This constellation of changes has created an oral anatomy that favors rapid plaque and calculus deposition, as well as gingival recession and cementum exposure in later stages of disease. These mechanically abrasive and bacteria laden surfaces, unsurprisingly, can elicit ulcerative and inflammatory changes in apposing oral mucosa (7).

We found a significant association between patient size and ultimate edentulism for any reason, with >70% of patients under 5 kg body weight reaching complete edentulism. There were no significant relationships between body weight and having any salvage exodontia or achieving salvage edentulism, so our study population likely has a large subset of very small dogs who develop contact lesions due to their substantial and often chronically untreated periodontitis. Although some sources (7) have questioned the earliest published claims of Maltese dogs being overrepresented (12, 13, 15), Maltese dogs (*n* = 7) and their crosses (*n* = 3) represent 24% of the present study population. The complete histopathologic descriptions in their original biopsy reports were reviewed, and none were supportive of primary eosinophilic oral mucosal disease. As none of the Maltese dogs in this study underwent salvage exodontic intervention solely for management of stomatitis, these cases may be more likely contact lesions secondary to advanced periodontal disease. However, Maltese and their crosses were both represented in the single-arm clinical trial for medical management of CCUS cases with cyclosporine and metronidazole (10). Based on the current body of literature, Maltese dogs may be overrepresented for both CL and CCUS. Further investigation is warranted to determine which consistent breed predispositions exist. Genetic testing may be particularly useful in pursuit of this goal.

Home oral hygiene interventions, though recommended for every canine stomatitis patient, were uncommonly employed. Only eight (19%) patients reportedly received any form of regular oral hygiene, with six (14%) having daily oral antiseptic rinses, three (7%) having their teeth brushed, and one (2%) utilizing dental wipes. Though the relative compliance rate for tooth brushing is higher in the present study than in a previous report (30), it is perhaps unsurprising that the generally low level of acceptance did not influence surgical or clinical outcome.

Preliminary diagnosis was not predictive of surgical or clinical outcome, and ten patients (24%) were reclassified from the original presumptive diagnosis during the study period. The initial classification as CL was questionable for 2 of 12 cases (17%) and the presumptive classification of CCUS was potentially incorrect for 10 of 30 cases (33%). Of these 10 presumptive CCUS patients, three were reclassified as CL due to resolution after periodontal therapy despite lacking radiographic evidence of periodontitis, and seven were reclassified as having no definitive diagnosis since oral mucosal disease was only partially responsive to therapy. At the end of the study period, 13 of 42 patients (31%) were confirmed as CL, 21 (50%) were confirmed as CCUS, and 8 (19%) could not be definitively diagnosed. CL cases showed high response rates to periodontal therapy (83%), while a subset of CCUS patients underwent salvage exodontia (20%) or immunosuppressive therapy (5%). In summary, the majority of canine cases with histologic diagnosis of lymphoplasmacytic stomatitis, including many diagnosed as CCUS, respond to periodontal disease management without the need for full-mouth extractions.

Dogs having any salvage extractions due to stomatitis and those rendered edentulous due to stomatitis were significantly more likely to have undergone multiple COHATs (*χ*^2^(1) = 8.40, *p* = 0.004; Fisher’s exact test *p* = 0.009). Similarly, patients undergoing any salvage extractions trended toward requiring rescue medical management between COHATs (*χ*^2^(1) = 4.748, *p* = 0.029; Fisher’s exact test *p* = 0.062), while those patients rendered edentulous due to stomatitis were significantly more likely to have required rescue medical management between COHATs (*χ*^2^(1) = 7.14, *p* = 0.008; Fisher’s exact test *p* = 0.012). These significant associations should still be interpreted cautiously, however. As was explicitly noted in the records of at least one partial responder, failure to return for additional treatment could represent a veterinary patient’s lack of agency and the client caregiver’s noncompliance. Although 50% of dogs only underwent a single COHAT episode, not returning for additional treatment could represent undiagnosed or untreated (rather than less severe) oral disease. Conversely, client or clinician preference to stage exodontic procedures may have increased the number of anesthetized assessments for a given patient.

Chronic immunosuppressive therapy was uncommonly employed during the study period, with only two (5%) patients being treated with chlorambucil. However, as the subdiscipline of oral medicine grows within veterinary dentistry, medical management of oral disease is increasingly described. Given the prevalence of underlying periodontitis, utilizing subantimicrobial doxycycline for matrix metalloproteinase antagonism could have meaningfully impacted the clinical course of some of the included patients (26). Application of the anti-inflammatory and antioxidant PENTO (pentoxifylline and tocopherol) protocol to CCUS patients has been attempted (25), but more promising still is a protocol of metronidazole and cyclosporine that has demonstrated efficacy in a single-arm clinical trial (10). Although salvage exodontia (i.e., extracting teeth at PD stages one and two) was an effective management strategy for 17% of cases in the present study, sufficient data exists to support offering immunomodulation as a less invasive treatment alternative for patients whose clinical course is most consistent with CCUS.

For the purposes of retrospective analysis, treatment outcome was broadly classified as complete, partial (i.e., persistent but improved lesions or clinical signs), or absent. No cases in the study had absent clinical response to treatment. In total, 81% patients achieved complete resolution of oral mucosal lesions and clinical signs during the study period: 62% solely via management of underlying periodontal disease, 17% with some degree of salvage exodontia, and one patient (2%) with chlorambucil prior to any salvage exodontia. Eight patients (17%) in the study had partial response to treatment (i.e., incomplete resolution of lesions); therefore, in the final classification, these cases had no definitive diagnosis. Four were lost to follow up after initial treatment recommendations were made. The remaining four had incomplete resolution of mucosal inflammation following salvage exodontia. These last four dogs represent those most likely to have had either refractory CCUS or another disease process such as immune-mediated disease, mucosal lymphoma, vasculitis, or other explanation for chronic stomatitis.

Limitations for the present study include those common to retrospective research, including heterogeneity in medical record keeping and patient management, as well as lack of standardized outcome reporting. As this is a retrospective study without a control (i.e., non-surgical) group, direct comparisons between surgical and long-term medical management cannot be made, nor can any potential synergistic benefit from multimodal management be inferred. Although 115 lesion site categories were noted across the included patient population, biopsy of each individual lesion was not required for inclusion. Multifocal lesions within a given patient were assumed to be from the same underlying disease process, which may not have been the case had biopsy sampling of every lesion been performed. This potentially explains why some cases presumptively classified as CL or CCUS had persistent mucosal lesions following therapy.

Further prospective research would benefit from objective measures such as the Canine Ulcerative Stomatitis Disease Activity Index (CUSDAI) to more systematically characterize disease at presentation as well as measuring response to therapy (8, 10). Medical records from other departments within the residency institution were utilized to obtain follow-up data for certain patients. Although complete records were required for inclusion, previous research has demonstrated that the accuracy of conscious oral examination findings can vary substantially based on clinician specialty (31); accordingly, more subtle lesions or clinical signs may have been missed. As the included data were collected over a decade, the very conceptual framework for the disease(s) of interest has evolved during the study period (i.e., CUPS giving way to CCUS and the need to recognize some contact lesions as distinct from CCUS as a clinical syndrome). This may have affected the manner in which individual clinicians approached management of these cases. An additional limitation concerns the potential for Type I error given the number of statistical comparisons performed. Because no multiplicity adjustment was applied, the probability of obtaining one or more statistically significant results by chance is increased. Accordingly, these findings should be regarded as exploratory and pending replication in larger cohorts.

**Figure 3:**
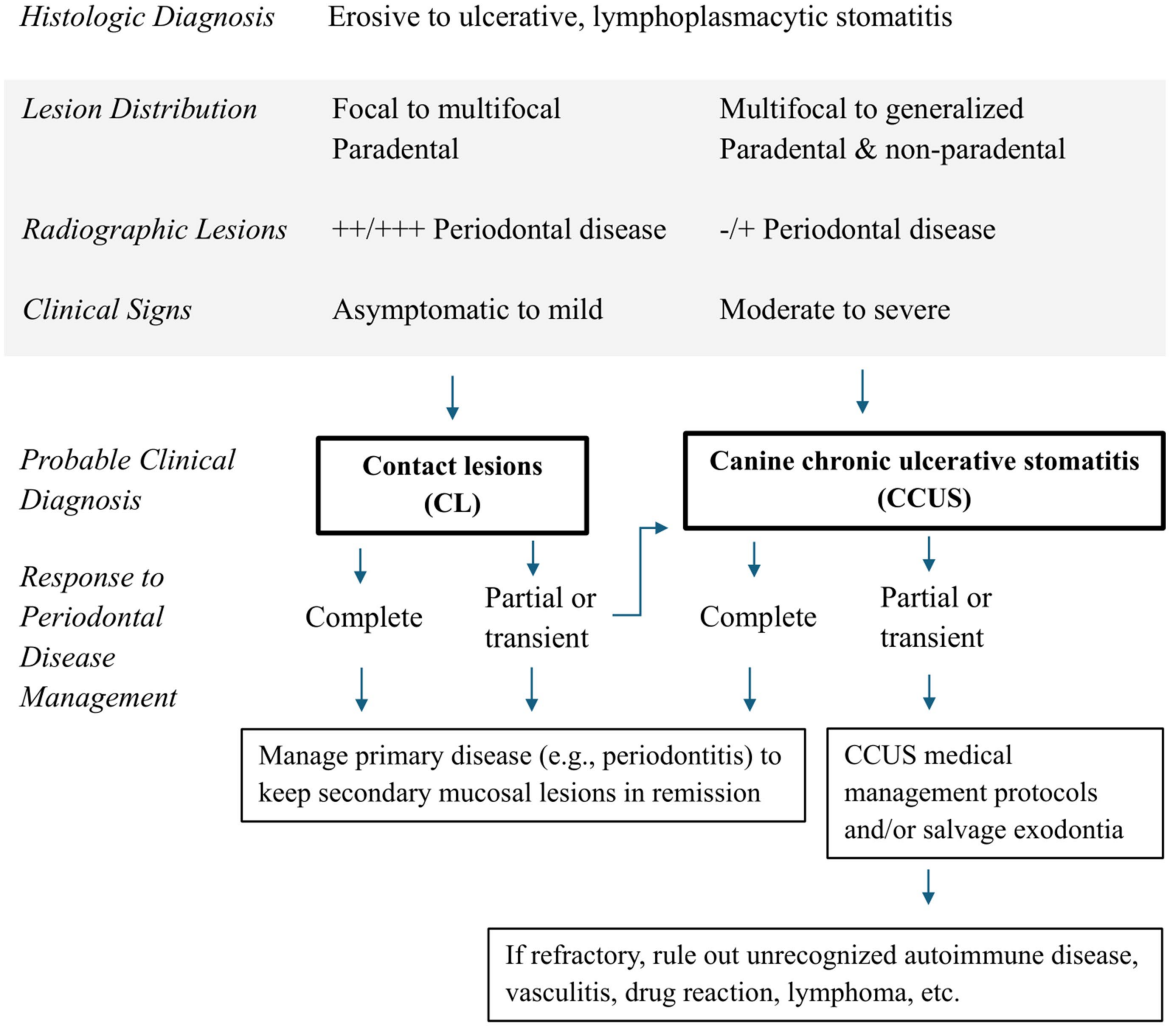
Proposed decision tree for chronic stomatitis in canine patients.

Appropriate management of lymphoplasmacytic stomatitis in canine patients requires careful consideration of historical, clinical, and histologic data (Figure 3). This is particularly important for CCUS and CL secondary to periodontitis since both are common yet have therapeutic and prognostic differences. If the periodontal disease burden of a given patient is commensurate with the severity and distribution of oral mucosal lesions, contact lesions should likely be prioritized initially. Management of the patient’s periodontal disease would be expected to concomitantly relieve secondary contact lesions, rendering either salvage exodontia or systemic immunomodulation excessive and unnecessary. Although the majority of patients in the present study achieved clinical control solely with treatment of underlying periodontal disease, medical management may be required to achieve remission in stomatitis patients refractory to surgical management. Consideration must also be given to unrecognized autoimmune disease, vasculitis, mucosal drug reactions, lymphoma, and other forms of stomatitis that can result from systemic disease yet present identically to either CL or CCUS (7, 24).

## Data Availability

The raw data supporting the conclusions of this article will be made available by the authors, without undue reservation.

## Footnotes

^1^Chi-square statistics were reported for consistency and interpretability, but Fisher’s exact tests were also conducted separately for each outcome given the small sample size (*n*=42; see Tables 1–5). Overall, Fisher’s exact test yielded comparable *p*-values thereby supporting the robustness of the reported chi-square results.

## References

[1] Hennet P. Chronic gingivostomatitis in cats: long-term follow-up of 30 cases treated by dental extractions. J Vet Dent. (1997) 14:15–21.

[2] Bellei E Dalla F Masetti L Pisoni L Joechler M. Surgical therapy in chronic feline gingivostomatitis (FCGS). Vet Res Commun. (2008) 32:231–4. doi: 10.1007/s11259-008-9153-8, 18685967

[3] Lommer MJ. Efficacy of cyclosporine for chronic, refractory stomatitis in cats: a randomized, placebo-controlled, double-blinded clinical study. J Vet Dent. (2013) 30:8–17. doi: 10.1177/089875641303000101, 23757820

[4] Jennings M Lewis J Soltero-Rivera M Brown D Reiter A. Effect of tooth extraction on stomatitis in cats: 95 cases (2000-2013). J Vet Dent. (2015) 246:654–60. doi: 10.2460/javma.246.6.654, 25719848

[5] Druet I Hennet P. Relationship between feline calicivirus load, oral lesions, and outcome in feline chronic gingivostomatitis (caudal stomatitis): retrospective study in 104 cats. Front Vet Sci. (2017) 4:209. doi: 10.3389/fvets.2017.00209, 29270412 PMC5724031

[6] Lobprise H Dodd J eds. Wiggs’s veterinary dentistry: principles and practice. 2nd ed. Hoboken, NJ: John Wiley & Sons, Inc. (2019).

[7] Murphy BG Bell CM Soukup JW. Veterinary oral and maxillofacial pathology. Hoboken, NJ: John Wiley & Sons, Inc. (2019).

[8] Anderson JG Peralta S Kol A Kass PH Murphy B. Clinical and histopathologic characterization of canine chronic ulcerative stomatitis. Vet Pathol. (2017) 54:511–9. doi: 10.1177/0300985816688754, 28113036

[9] Anderson JG Kol A Bizikova P Stapelton BP Ford K Villarreal A . Immunopathogenesis of canine chronic ulcerative stomatitis. PLoS One. (2020) 15:e0227386. doi: 10.1371/journal.pone.0227386, 31923271 PMC6953816

[10] Ford KR Anderson JG Stapleton BL Murphy BG Kumar TKS Archer T . Medical management of canine chronic ulcerative stomatitis using cyclosporine and metronidazole. J Vet Dent. (2023) 40:109–24. doi: 10.1177/08987564221148755, 36650996

[11] Merillat L. Animal dentistry and diseases of the mouth, vol. 1. London, UK: Bailliere, Tindall and Cox (1905).

[12] Harvey CE Peter E. Small animal dentistry. St. Louis, MO: Mosby (1993).

[13] Carmichael D. Dental corner: diagnosing and treating chronic ulcerative paradental stomatitis DVM360 (2004). Available online at: https://www.dvm360.com/view/dental-corner-diagnosing-and-treating-chronic-ulcerative-paradental-stomatitis

[14] Boutoille F Hennet P. Maxillary osteomyelitis in two Scottish terrier dogs with chronic ulcerative paradental stomatitis. J Vet Dent. (2011) 28:96–100. doi: 10.1177/089875641102800206, 21916373

[15] Niemiec B ed. Small animal dental, oral and maxillofacial disease: a color handbook. Boca Raton, FL: Manson Publishing/The Veterinary Press; CRC Press (2010).

[16] Joffe D Allen A. Ulcerative eosinophilic stomatitis in three cavalier king Charles spaniels. J Am Anim Hosp Assoc. (1995) 31:34–7. doi: 10.5326/15473317-31-1-34, 7820762

[17] German AJ Holden DJ Hall EJ Day MJ. Eosinophilic diseases in two cavalier king Charles spaniels. J Small Anim Pract. (2002) 43:533–8. doi: 10.1111/j.1748-5827.2002.tb00026.x, 12489741

[18] Mendelsohn D Lewis JR Scott KI Brown DC Reiter AM. Clinicopathological features, risk factors and predispositions, and response to treatment of eosinophilic oral disease in 24 dogs (2000-2016). J Vet Dent. (2019) 36:25–31. doi: 10.1177/0898756419834785, 31138045

[19] Banovic F Olivry T Bazzle L Tobias JR Atlee B Zabel S . Clinical and microscopic characteristics of canine toxic epidermal necrolysis. Vet Pathol. (2015) 52:321–30. doi: 10.1177/0300985814537530, 24907312

[20] Declercq J. Suspected wood poisoning caused by Simarouba amara (marupá/caixeta) shavings in two dogs with erosive stomatitis and dermatitis. Vet Dermatol. (2004) 15:188–93. doi: 10.1111/j.1365-3164.2004.00377.x, 15214956

[21] Bizikova P Linder KE Anderson JG. Erosive and ulcerative stomatitis in dogs and cats: which immune-mediated diseases to consider? J Am Vet Med Assoc. (2023) 261:S48–57. doi: 10.2460/javma.22.12.0573, 37059419

[22] Arzi B Anderson JG Verstraete F. Oral manifestations of systemic disorders in dogs and cats. J Vet Clin Stud. (2008) 1:112–24.

[23] Henry P Perry AJ Mackenzie DP. Recurrent ulcerative necrotising stomatitis in two dogs with concurrent steroid-responsive chronic rhinitis and suspected underlying oral vasculitis. Vet Rec Case Rep. (2022) 10:488. doi: 10.1002/vrc2.488

[24] Anderson JG. Canine oral lesions: a decision-tree approach to ulcers, leukoplakia, and pigmented lesions. J Am Vet Med Assoc. (2023) 261:S62–9. doi: 10.2460/javma.23.05.0294, 37699542

[25] Gracis M Reiter A Ordeix L. Management of selected non-periodontal inflammatory, infectious and reactive conditions In: Reiter A Gracis M, editors. BSAVA manual of canine and feline dentistry and Oral surgery. 4th ed. Gloucester, UK: British Small Animal Veterinary Association (2018). 172–95.

[26] Kim SE Hwang SY Jeong M Lee Y Lee ER Park YW . Clinical and microbiological effects of a subantimicrobial dose of oral doxycycline on periodontitis in dogs. Vet J. (2016) 208:55–9. doi: 10.1016/j.tvjl.2015.10.006, 26639830

[27] Algorta A Turini G Martín Eguren J María Grande A K Yaneselli1 Benavides U . Local treatment of canine chronic ulcerative stomatitis using adipose tissue derived mesenchymal stem cells- a case report. Veterinarski Arhiv. (2022) 92:359–67. doi: 10.24099/vet

[28] Hermanson JW DeLahunta Alexander Evans HE. Miller and Evans’ anatomy of the dog. 5th ed. St. Louis, MO: Saunders (2019).

[29] Nanci A ed. Ten Cate’s Oral histology: Development, structure, and function. 9th ed. St. Louis, MO: Elsevier (2017).

[30] Enlund KB Brunius C Hanson J Hagman R Höglund OV Gustås P . Dental home care in dogs- a questionnaire study among Swedish dog owners, veterinarians and veterinary nurses. BMC Vet Res. (2020) 16:90. doi: 10.1186/s12917-020-02281-y, 32188446 PMC7081671

[31] Soukup JW Hetzel S Paul A. Classification and epidemiology of traumatic dentoalveolar injuries in dogs and cats: 959 injuries in 660 patient visits (2004-2012). J Vet Dent. (2015) 32:6–14. doi: 10.1177/089875641503200101, 26197685

